# Efficacy of a novel device for cryoprevention of oral mucositis: a randomized, blinded, multicenter, parallel group, phase 3 trial

**DOI:** 10.1038/s41409-021-01512-6

**Published:** 2021-11-03

**Authors:** Java Walladbegi, Roger Henriksson, Björn Tavelin, Anncarin Svanberg, Gunnar Larfors, Martin Jädersten, Fredrik Schjesvold, Aram Mahdi, Karin Garming Legert, Douglas E. Peterson, Mats Jontell

**Affiliations:** 1 Department of Oral Medicine and Pathology, Institute of Odontology, The Sahlgrenska Academy, University of Gothenburg, Gothenburg, Sweden; 2 Department of Radiation Sciences-Oncology, Umea University, Umea, Sweden; 3 Department of Medical Sciences Hematology, Uppsala University, Uppsala, Sweden; 4 Department of Hematology M64, Karolinska University Hospital, Huddinge, Stockholm, Sweden; 5 Oslo Myeloma Centre, Department of Hematology, Oslo University Hospital, Oslo, Norway; 6 K.G. Jebsen Centre for B-cell Malignancies, University of Oslo, Oslo, Norway; 7 Division of Oral Diagnostics and Rehabilitation, Department of Dental Medicine, Karolinska Institutet, Stockholm, Sweden; 8 Department of Oral Health and Diagnostic Sciences, School of Dental Medicine, UConn Health, Farmington, Connecticut USA

**Keywords:** Myeloma, Lymphoma, Randomized controlled trials

## Abstract

Cryoprevention (CP) using ice (IC) is an effective strategy to prevent chemotherapy-induced oral mucositis (OM). However, the use of IC may cause adverse reactions and requires water of safe quality to minimize risk of serious infections. This randomized, blinded, parallel group, phase 3 trial was conducted in five Scandinavian centers. Eligible patients were diagnosed with multiple myeloma or lymphoma, scheduled to receive conditioning with high-dose chemotherapy prior to autologous hematopoietic stem cell transplantation (ASCT). Patients were assigned to cooling with IC or a novel intraoral cooling device (ICD). The primary outcome was the highest OM score during the study period, expressed as peak value on the Oral Mucositis Assessment Scale (OMAS–total). When the entire study population (*n* = 172) was analyzed for peak OMAS–total, the two cooling methods were equally effective. However, when the lymphoma group was analyzed separately, the ICD significantly reduced the peak OMAS–total score to a greater extent compared to IC (*x̄* ± SD; 1.77 ± 1.59 vs. 3.08 ± 1.50; *p* = 0.047). Combined with existing evidence, the results of the present trial confirm that CP is an effective method to prevent OM. ClinicalTrials.gov. NCT03203733.

## Introduction

Oral mucositis (OM) affects up to 80% [1] of all patients conditioned with high-dose chemotherapy in conjunction with hematopoietic stem cell transplantation (HSCT) [2, 3]. In aggregate, OM results in adverse clinical sequelae including increased need for analgesics, parenteral nutrition, interruptions or alterations of effective cancer treatment modalities, extended hospital visits [4, 5], and increased healthcare costs [3, 4, 6, 7]. Once established, OM may serve as a portal of entry for endogenous flora or waterborne pathogens [5]. This may increase the risk for sepsis and a fatal outcome [5, 8–10].

Cryotherapy (CT) using ice chips (ICs) has been used prophylactically to reduce the onset and duration of chemotherapy-induced OM [11]. Promotion of vasoconstriction resulting in reduced delivery of chemotherapeutic agents to the oral mucosa continues to be viewed as the most likely mechanism of action [12]. The preservation of the tissue could also be attributed to a decreased metabolic activity in the basal epithelial cell layer resulting in lower exposure to cytotoxic drugs [13, 14].

The 5-year overall survival rate is significantly higher in patients using CT compared to standard oral care [15]. However, despite favorable observations, the use of IC as a preventive method in clinical practice is limited [16]. One explanation for this is that IC may entail adverse reactions such as chills, nausea, and shooting with sharp pain in the teeth, which could influence patient tolerability, leading to poorer adherence [2, 17, 18]. In addition, concerns have been raised that water to produce ICs may contain microorganisms in concentrations that could increase risk of serious infections in immunocompromised oncology patients [19].

To our knowledge, this is the first randomized clinical trial comparing conventional ice therapy with an intraoral cooling device (ICD). This study builds upon the evidence established for CT over the past three decades, while incorporating contemporary novel cooling technology in order to further enhance clinical outcomes.

## Subjects and methods

### Trial design and participants

The study was carried out in five university hospitals in Sweden and Norway as follows: Uppsala University Hospital, Karolinska University Hospital, Linköping University Hospital, Örebro University Hospital, and Oslo University Hospital. Ethical approval was obtained by the Swedish Ethical Review Authority, Sweden (Reference number 586-15), and the Regional Committee for Medical and Health Research Ethics, Oslo, Norway (Reference number 2018/1653). Investigators included patients (≥18 years) confirmed with multiple myeloma or lymphoma and scheduled to receive high-dose conditioning chemotherapy prior to autologous stem cell transplantation (ASCT). Patients were eligible given that the investigator considered them as appropriate candidates for high-dose melphalan (multiple myeloma) or high-dose regimen with BEAC (carmustine, cytarabine, etoposide, cyclophosphamide) and BEAM (carmustine, cytarabine, etoposide, melphalan) (lymphoma). Exclusion criteria were involvement in other trials, which, according to the investigator, could interfere with the outcomes of this trial, or if posttransplantation follow-ups occurred at hospitals outside the regions of the study centers. Prior to enrollment, written informed consent was obtained from all patients and they were further informed about their rights to withdraw consent to participate in the study at any given time without reprisal or stating reason for withdrawal. The trial protocol and statistical analysis plan are available online (Supplement 1).

### Randomization

Eligible patients were randomly assigned 1:1 to cooling with ICs or ICD by means of randomization lists with the use of permuted blocks stratified according to diagnosis. Randomly varying block sizes (2, 4, or 6), where 1, 2, or 3 tests (*t*) were distributed in sequences along with 1, 2, or 3 comparisons (*c*). All centers were blinded to the size of the blocks. Randomization lists were generated by Karolinska Trial Alliance. The responsible physician and the medical staff in charge of the cooling procedures did not participate in the assessment of the outcomes. The dental staff involved in the clinical outcome of OM were masked to the interventions and patients were strictly informed not to mention to which of the two interventions they had been assigned. Statistical analyses were conducted by a statistician who remained masked throughout the course of the study.

### Interventions

Each cooling session commenced 30 min prior to the chemotherapy infusion (Supplement 2) and continued for 30 min after the infusion was completed. One cooling session corresponding to 1.5 h was assigned per patient in the myeloma group, whereas the lymphoma group was subjected to the following cooling modalities: BEAC: 2 cooling sessions per day of 1.5–3.5 h per session for 5 consecutive days or BEAM: 1–2 cooling sessions per day of 1–2 h per sessions for 6 consecutive days.

Each study site manufactured IC from tap water. The IC temperature was approximately −0.5 °C upon exposure. Patients were informed to insert an ounce of ice and move the IC around in the mouth. They were also briefed to rinse the melted ice slurry that was obtained before it was swallowed or expectorated. The ICD (Cooral® Mouth device) was provided by BrainCool AB, Lund, Sweden. The ICD is a single-use device and it was available in two sizes (medium and large). It consists of a closed conduit system with continuously circulating water delivered by a portable thermostat unit (Cooral® System). Water temperature of 8 °C (±2 °C) was used as default settings throughout the trial [17, 20].

### Assessments

Degree of OM was assessed at the buccal and palatal mucosae, lips, floor of the mouth, tongue, and gingiva, using the Oral Mucositis Assessment Scale (OMAS; graded 0–3 for ulceration and 0–2 for erythema; Supplement 3). Zero corresponds to healthy oral mucosa and 1 ≤ 1 cm^2^; 2 = 1–3 cm^2^; 3 ≥ 3 cm^2^ corresponds to the total area of ulcerations. The corresponding figures for erythema are 1 = mild; 2 = severe. Thus, the assessment generated both an average for OMAS–ulceration (0–3) and OMAS–erythema (0–2), and the total average score (0–5). Oral medicine specialists were trained to assess the degree of OM. Intraclass correlation coefficient (ICC) was performed on a sample data set to ascertain inter-rater reproducibility agreement between the dental staff for OM with OMAS; ICC = 0.994 [95% confidence interval (95% CI) 0.984–0.999]; *p* < 0.0001. Each patient was assessed three times a week, beginning at admission and continuing until discharge or until day +28 after ASCT. The highest OM score during the time in care, expressed as peak OMAS–total was used to perform the statistical analysis.

Following each cooling session, tolerability was gathered using a study-specific questionnaire (evaluation survey; Supplement 4). The questionnaire comprised the following response alternatives (1 = Not at all painful; 2 = Slightly painful; 3 = Rather painful; 4 = Painful; 5 = Very painful; 6 = Very, very painful; 7 = Extremely painful). However, as none of the response alternatives (5–7) were reported, incidence of problems (1–2) were grouped and compared with incidence of (3–4). In the lymphoma group, the highest reported value during the 5 (BEAC) or 6 (BEAM) cooling days was used in the statistical analysis. Prior to the study, all questions and response alternatives were verified by an independent group of patients (*n* = 5).

Patient-reported oral pain due to OM was assessed using the Numeric Pain Rating Scale (NPRS) with the extremes graded on a 0–10 scale. Data on patient-reported oral pain was collected daily beginning at admission and continuing until discharge or day +28 after ASCT. The peak value for patient-reported oral pain during the time in care was utilized for statistical analysis.

This study also included a validated quality of life (QoL) instrument (the functional assessment of cancer therapy - general (FACT–G), version 4; Supplement 5), which was completed once at admission and again at discharge. Other variables assessed during the course of this study were as follows: total parenteral nutrition (TPN); hospital days; dose of opioid analgesics; C-reactive protein (CRP); weight; leukocyte plasma concentration; absolute neutrophil count (ANC); serum Albumin (s-Albumin); and core body temperature.

### Outcomes

The primary outcome was (i) OM, defined as peak OMAS–total. The secondary outcomes were as follows: (ii–a) degree of tolerability and (ii–b) patient-reported oral pain, defined as NPRS ≥ 3. The tertiary outcomes were as follows: (iii–a) QoL at admission and discharge, using FACT–G, version 4; (iii–b) number of days with TPN; (iii–c) number of hospital days; (iii–d) total dose of opioid analgesics converted to morphine (mg); (iii–e) peak CRP (mg/L); (iii–f) maximum weight loss, defined as initial value minus the lowest value (kg); (iii–g) number of days from transplantation to bone marrow engraftment, defined as ANC > 1.0 × 10^9^ cells/L; (iii–h) maximum drop for s-Albumin, defined as initial value minus lowest value (g/L); and (iii–i) maximum temperature increase, defined as highest value minus the initial value (°C).

Adverse events reported by the patients for the two interventions were assessed with the study-specific questionnaire (Supplement 4). Any serious adverse events were documented and shared with all study sites.

### Sample size and power calculation

A sample size of at least 90 patients per group would give a power of 80% to discover an average difference of at least 0.42 OMAS–total units [21]. The analysis was calculated based on the SD for OMAS–total being one in both groups and the use of the independent samples *t*-test with an *α*-significance level of 0.05, employing G*power version 3.1.9.4 (University of Düsseldorf, Düsseldorf, Germany).

### Statistical analysis

The primary outcome (i) was studied in a multiple linear regression model. Treatment group, type of cancer, and center were used as fixed explanatory variables. The initial model further included interaction between treatment and type of cancer, as well as interaction between treatment and center. If the interaction effects were not significant, they were excluded from the final model. Differences in mean peak OMAS–total between the treatment groups were tested with the Mann–Whitney’s *U*-test. The probability of free peak OMAS–total ≥ 3 for the two intervention groups, considering each diagnosis separately, were estimated using the Kaplan–Meier method and the log-rank test. The secondary outcomes (ii–a to b) were analyzed non-parametrically by use of Mann–Whitney’s *U*-test. The tertiary outcomes (iii–a to i) were analyzed using independent t-test or Mann–Whitney’s *U*-test. The difference in the number of adverse events for the two interventions were analyzed statistically using Pearson’s *χ*^2^-test or Fisher’s exact test.

Primary and safety analyses were performed by intention-to-treat. The statistical analyses were employed using the IBM SPSS Statistics software package (IBM SPSS Statistics version 25, IBM, Armonk, NY). A *p*-value ≤ 0.05 was considered statistically significant.

## Results

From 12 June 2017 to 12 November 2019, 182 eligible patients with multiple myeloma (*n* = 156; 85.7%) or lymphoma (*n* = 26; 14.3%) were included and randomly assigned (1:1) to cooling with IC (*n* = 92; 50.5%) or ICD (*n* = 90; 49.5%). Patient characteristics and demographics at baseline are presented in Table 1.

**Table 1:** Characteristics and demographics at baseline.

	Ice chips (ICs)	Intraoral cooling device (ICD)
	Comparison group (*n* = 92) [%]	Test group (*n* = 90) [%]
Sex		
Male	53 [58%]	59 [66%]
Female	39 [42%]	31 [34%]
Age, years (*x̄* ± SD)	61 ± 8	59 ± 9
Diagnoses		
Multiple myeloma	79 [85.9%]	77 [85.6%]
Conditioning chemotherapy		
Melphalan 140 mg/m^2^	2 [2.5%]	2 [2.6%]
Melphalan 200 mg/m^2^	77 [97.5%]	75 [97.4%]
Lymphoma	13 [14.1%]	13 [14.4%]
Non-Hodgkin’s^a^	12 [92.3%]	8 [61.5%]
Mantle Cell	5 [41.6%]	3 [37.5%]
Diffuse large B cell	2 [16.7%]	5 [62.5%]
Follicular	3 [25.0%]	-
High-grade B cell	2 [16.7%]	-
Hodgkin’s^a^	-	2 [15.4%]
T cell	1 [7.7%]	1 [7.7%]
Other atypical subtypes	-	2 [15.4%]
Conditioning chemotherapy		
BEAC	12 (13.0%)	11 [12.2%]
BEAM	1 (1.1%)	2 [2.2%]
ASCT		
I	70 [76.1%]	77 [85.5%]
II	22 [23.9%]	11 [12.2%]
III	-	2 [2.3%]

*ASCT* autologous hematopoietic stem cell transplantation (the Roman numerals refer to the number of ASCTs), *x̄* mean.

High-dose chemotherapy regimens for lymphoma: BEAC (carmustine; cytarabine; etoposide; cyclophosphamide) and BEAM (carmustine; cytarabine; etoposide; melphalan).

^a^B-cell lymphomas.

Uppsala University Hospital included patients with myeloma (*n* = 43/69; 62.3%) or lymphoma (*n* = 26/69; 37.7%). In the other four study sites, only patients with multiple myeloma were included.

**Fig. 1:**
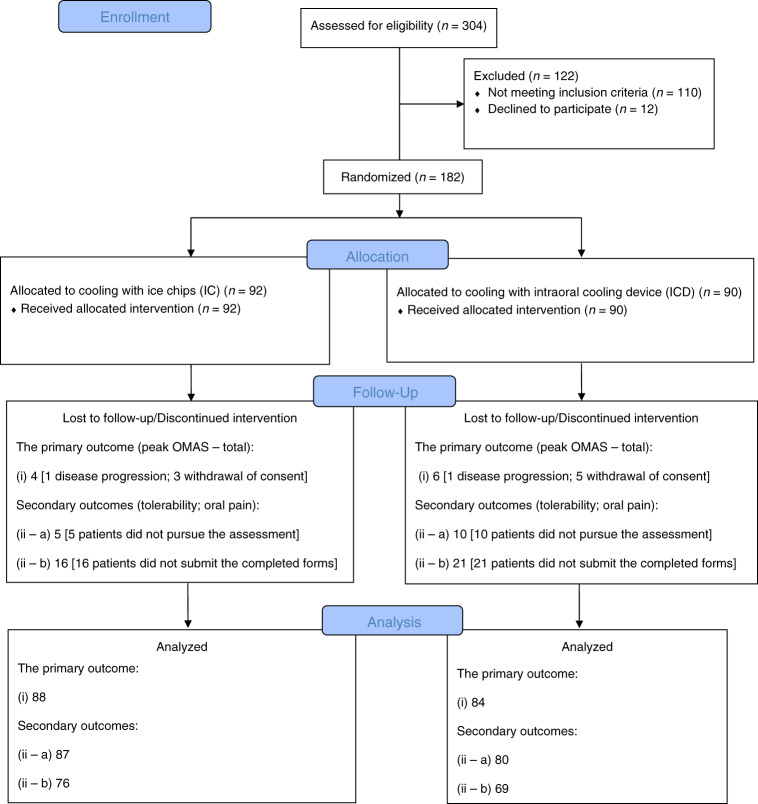
Flow diagram. In total, ten patients (*n* = 10) dropped out of the enrolled study sample (*n* = 182; IC = 92; ICD = 90) related to the primary outcome (*i*) [*n* = 172; IC = 88; ICD = 84]. Two patients (*n* = 2; 1 in each intervention arm) did not continue the study due to fatal outcome related to disease progression. Eight patients (*n* = 8; 3 in the IC group and 5 in the ICD group) withdrew their consent to participate in the study. For the secondary outcome tolerability (ii–a) [*n* = 167; IC = 87; ICD = 80], 15 (*n* = 15; 5 in the IC group and 10 in the ICD group) patients were not able to pursue the assessment; for the secondary outcome patient-reported oral pain (ii–b) [*n* = 145; IC = 76; ICD = 69], 37 (*n* = 37; 16 in the IC group and 21 in the ICD group) did not submit the completed forms. IC, ice chips; ICD, intraoral cooling device.

The reasons for discontinuing the study were either a fatal outcome related to disease progression (*n* = 2), one in each intervention arm, or withdrawal of the consent (*n* = 8: IC = 3; ICD = 5). Regarding tolerability, 15 patients (IC = 5; ICD = 10) were not able to pursue the assessment. However, a detailed analysis of this lack of tolerability assessments did not reveal any correlation to the two interventions. As for patient-reported oral pain scales, these forms were collected when OM had completely resolved or +28 days after ASCT. The fully enrolled sample assigned to each intervention arm and dropouts related to the primary and secondary outcomes are presented in the flow diagram (Fig. 1).

### Primary outcome

A multiple linear regression was calculated to predict peak OMAS–total based on treatment group, type of cancer (Table 2), and study center. Peak OMAS–total decreased with the coefficient (−0.297 units) more for ICD than for IC and increased with the coefficient (0.923 units) for lymphoma compared to multiple myeloma. The latter reached a statistical significance; *p* = 0.007.

**Table 2:** Multiple linear regression model.

Peak OMAS–total	Coefficient	*p*-Value	95% Confidence interval
Treatment group	−0.297	0.158	−0.712 to 0.117
Type of cancer	0.923	0.007	0.252 to 1.594
(Constant)	1.649	<0.001	1.190 to 2.107

Dependent variable: peak OMAS–total; independent variables: treatment group and type of cancer.

When the total population (*n* = 172: IC = 88; ICD = 84) (multiple myeloma: *n* = 146; lymphoma: *n* = 26) was analyzed, OM of any grade was found in 44.2% of the patients (*n* = 76/172). OM was found to be considerably higher for the patients with lymphoma 80.8% (*n* = 21/26) compared with the myeloma patients 37.7% (55/146). Severe OM (OMAS ≥ 3) was observed in 19.3% of the entire cohort (*n* = 33/172), 50.0% of the lymphoma group (*n* = 13/26), and 13.7% of the myeloma group (*n* = 20/146).

**Fig. 2:**
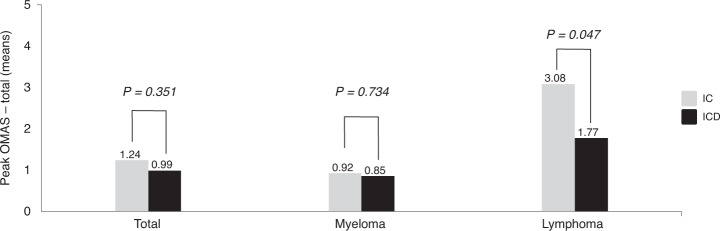
Peak OMAS-total. Peak OMAS–total (mean) for the total population, myeloma group, and the lymphoma group following cooling with ice chips (ICs) or the intraoral cooling device (ICD).

**Fig. 3:**
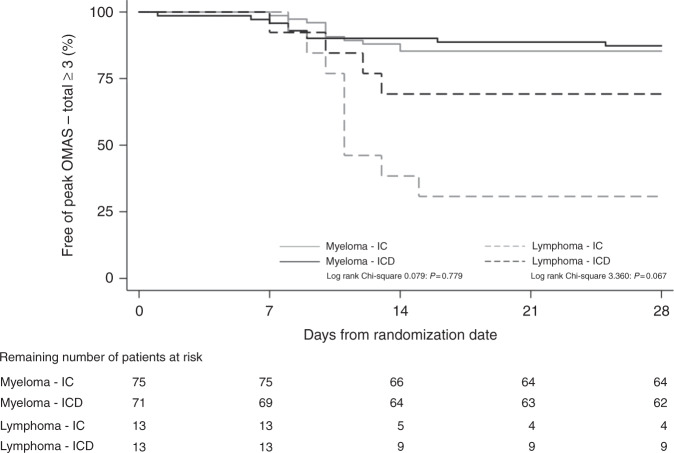
Kaplan-Meier diagram. Number of patients at risk of severe oral mucositis, defined as peak OMAS–total ≥ 3 (%), following conditioning chemotherapy.

Furthermore, regarding the entire study cohort, no statistically significant difference was found between IC and ICD in prevention of chemotherapy-induced OM, i.e., peak OMAS–total (*x̄* ± SD; 1.24 ± 1.61 vs. 0.99 ± 1.47; *p* = 0.351). However, a statistically significant difference was found within the lymphoma group between IC and ICD (*x̄* ± SD; 3.08 ± 1.50 vs. 1.77 ± 1.59; *p* = 0.047). The corresponding figures for myeloma were (*x̄* ± SD; 0.92 ± 1.41 vs. 0.85 ± 1.41; *p* = 0.734; Fig. 2). The Kaplan–Meier diagram (Fig. 3) shows that severe OM, i.e., OMAS–total ≥ 3 mainly occurred during the second week after ASCT. The figure also supports the superiority of ICD to prevent severe OM expressed as peak OMAS–total ≥ 3 in lymphoma patients.

### Secondary outcomes

**Fig. 4:**
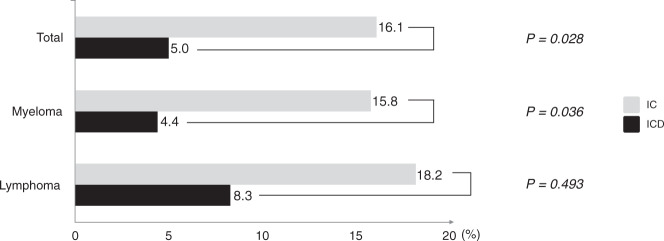
Percentage of patients reporting discomfort after cooling with ice chips (ICs) or the intraoral cooling device (ICD). Data are shown for the total population (*n* = 167: IC = 14/87; ICD = 4/80), myeloma group (*n* = 144: IC = 12/76; ICD = 3/68), and the lymphoma group (*n* = 23: IC = 2/11; ICD = 1/12).

In terms of tolerability (Fig. 4) for the entire population (*n* = 167: IC = 87; ICD = 80), the two levels “not at all painful” and “slightly painful” were compared as a group with the group of the two higher levels “rather painful” and “painful.” In the group using the ICD, 5.0% (*n* = 4/80) reported discomfort, whereas the comparable score for the patients randomized to IC was 16.1% (*n* = 14/87). This difference reached statistical significance (odds ratio (OR) = 0.274 [95% CI 0.086–0.873]; *p* = 0.028). When the subgroups, i.e., multiple myeloma (*n* = 144: IC = 76; ICD = 68) and lymphoma (*n* = 23: IC = 11; ICD = 12) were analyzed separately, the corresponding figures for ICD and IC were 4.4% (*n* = 3/68) vs. 15.8% (*n* = 12/76) and 8.3% (*n* = 1/12) vs. 18.2% (*n* = 2/11), respectively. The statistical analyses supported a higher degree of tolerability in the myeloma group when ICD was compared with IC (OR = 0.246 [95% CI 0.066–0.914]; *p* = 0.036), whereas no statistically significant difference was observed between the two interventions in the lymphoma group (OR = 0.409 [95% CI 0.032–5.276]; *p* = 0.493).

Regarding the pain score, 145 completed forms were obtained for the myeloma and lymphoma groups combined (IC = 76; ICD = 69). Clinically significant discomfort (NPRS ≥ 3) was reported by 23.2% (*n* = 16/69) in the ICD group and 36.8% of the patients (*n* = 28/76) using ICs. This difference of 13.6 percentage points did not reach a statistical significance (OR = 0.518 [95% CI 0.250–1.072]; *p* = 0.076).

### Tertiary outcomes

No statistically significant difference was found between the two interventions for any of the tertiary outcomes (iii–a to i).

Concomitant registrations for patient-reported adverse events and serious adverse events for the two subgroups combined are presented in Table 3. A statistically significant difference was found between two cooling procedures for the following four adverse events (IC vs. ICD): numbness (10.3% vs. 2.5% *p* = 0.041), teeth hypersensitivity (19.5% vs. 6.3% *p* = 0.011), nausea (12.6% vs. 3.8% *p* = 0.038), and difficulties swallowing during the cooling procedure (2.3% vs. 20.0% *p* < 0.001). Furthermore, no serious adverse event was reported.

**Table 3:** Adverse events.

Multiple myeloma and lymphoma	Ice chips (IC = 87)	Intraoral cooling device (ICD = 80)	*p*-Value
Adverse events (*n*) [%]			
Chills	24 [27.6%]	13 [16.3%]	0.078
Numbness	9 [10.3%]	2 [2.5%]	**0.041**
Bad taste	6 [6.9%]	2 [2.5%]	0.281
Headache	2 [2.3%]	2 [2.5%]	1.000
Teeth hypersensitivity	17 [19.5%]	5 [6.3%]	**0.011**
Oral soreness	8 [9.2%]	5 [6.3%]	0.478
Poor fit ^ICD^	-	16 [20.0%]	-
Nausea	11 [12.6%]	3 [3.8%]	**0.038**
Vomiting sensation	5 [5.7%]	6 [7.5%]	0.648
Difficulties swallowing	2 [2.3%]	16 [20.0%]	**<0.001**
Rubbing discomfort^ICD^	-	24 [30.0%]	-
Other discomforts	12 [13.8%]	17 [21.3%]	0.204
Serious adverse events (*n*) [%]	-	-	-

The number (*n*) and proportion [%] of adverse events and serious adverse events reported for ice chips (ICs) and intraoral cooling device (ICD) in myeloma and lymphoma patients. The two response alternatives superscripted with ICD were only available in the cooling sessions with the ICD.

## Discussion

The ICD proved to further enhance the efficacy of conventional ICs in prevention of OM in patients with lymphoma, whereas efficacy results were comparable between the two cooling methods in the myeloma group. Tolerability was higher for ICD compared to IC in the myeloma group. Higher tolerability of the ICD was also observed in the lymphoma patients, although was not statistically significant. The collective tolerability data were in accordance with our previous study on healthy volunteers [17].

Cryoprevention (CP) is not part of a global routine clinical practice for patients who are receiving high-dose chemotherapy prior to HSCT and in particular for patients subjected to multi-day conditioning regimens [16]. One concern is the potential risk of contaminated water. Although not specifically studied in this clinical trial, it is biologically plausible to suggest that drinking water may contain facultative pathogenic microorganisms and it should be emphasized that there is a substantial risk of infections in immunodeficient patients in selected centers [5, 19, 22–24]. This risk is eliminated using the ICD.

Pain was reported to a greater extent in the IC group than in the ICD group, although no statistically significant difference was found between the two interventions. The lower incidence of oral pain in the ICD group concurred with the superior effects of the ICD for tolerability and reduced severe OM in the lymphoma group. Intractable oral pain is most likely a consequence of erythema or ulcers [5, 25]. Physical pain related to the development of OM has attained the highest score followed by physical disability and psychological discomfort [26].

Considering the serious outcomes of OM, it is not surprising that resource utilization is increased significantly among the affected patients. However, the economic consequences of OM are often underappreciated. The incremental cost of OM-associated hospitalization among stem cell recipients is high, exceeding USD 70,000 for patients who develop ulceration and USD 375,000 among those with severe OM [5, 6, 27].

In their most recent publication [3], MASCC/ISOO recommended the use of intravenously administrated keratinocyte growth factor-1 (KGF-1) for the prevention of OM. However, despite statistically significant differences, about 60% of the patients treated with KGF-1 still experienced severe OM [28]. In 2016, the marketing authorization for KGF-1 has been withdrawn by the European Medicines Agency.

CP and photobiomodulation (PBM) combined have been compared to PBM alone for the reduction of OM. The combination of CP with PBM was superior to PBM alone in reducing the severity of OM [29]. However, it is noteworthy that a study arm with only CP was not included. Thus, the contribution of PBM is unknown from this study. CP is recommended to be used to alleviate OM in patients receiving bolus 5-fluorouracil chemotherapy and in patients who are receiving high-dose melphalan in preparation for HSCT [2]. Thus, CP has a broader indication profile compared to PBM; the latter only recommended for patients conditioned with high‐dose chemotherapy in preparation for HSCT.

The present study has limitations. First, this randomized trial was not primarily designed for the secondary endpoint to compare the tolerability of the ICD and IC, as no crossover design was possible to conduct. Furthermore, the difficulties to swallow when the ICD was used may have been prevented by a more appropriate adoption to different sizes of the oral cavity. However, despite these limitations, a higher degree of tolerability was reported by the total patient population when ICD was compared with IC.

Second, the small number of patients with lymphoma, who were included in the study, and that these patients were enrolled from one study site, Uppsala, which primarily uses BEAC as conditioning for lymphoma. BEAC results in less pronounced mucositis compared to the more commonly used BEAM [30]. It is conceivable that the statistically significant reduction in OM with the ICD would have been even more pronounced in lymphoma patients receiving BEAM.

A third limitation was the lack of a triple blind Randomized Controlled Trial (RCT) design. However, both the dental staff involved in the clinical outcome of OM and the statistician were blinded to the interventions. In addition, patients were strictly informed not to mention which of the two cooling methods to which they had been assigned.

In conclusion, the present study emphasizes the importance of the strategy of CP to prevent OM. The conventional cooling method of IC was shown to be further improved using the ICD in prevention of OM for lymphoma patients. An estimated number needed to treat was calculated to 2.6, which means that this number of lymphoma patients had to be treated with IC to prevent one case of OM in comparison with ICD. Moreover, as the lymphoma patients received cooling for up to 6 h, the study provides clinical evidence that longer cooling sessions are tolerated. This may imply that the ICD can be used to prevent OM in other cancer groups than those included in this study, e.g., breast cancer patients where the infusion time of the cytotoxic drugs lasts for a shorter time compared to the lymphoma group.

## Supplementary information

Supplement 1

Supplement 2

Supplement 3

Supplement 4

Supplement 5
