# Supplementary material for: Efficacy of a novel device for cryoprevention of oral mucositis: a randomized, blinded, multicenter, parallel group, phase 3 trial
Source: Bone Marrow Transplant. 2021 Nov 3;57(2):191–7. doi: 10.1038/s41409-021-01512-6 (PMC8821013; doi:10.1038/s41409-021-01512-6)

19th October 2018
Version 6 (Final)

Cryoprevention of chemotherapy-induced oral mucositis after autologous stem cell transplantation, a randomized study

Protocol, final version
Revised: 19th October 2018

Java Walladbegi · Bengt Furberg · Anncarin Svanberg · Martin Gellerstedt · Mats Jontell

Background 1

Aim 1

Material and Method 2
Design 2
Selection 2
Inclusion criteria 2
Exclusion criteria 2
Randomization 2
Power 3
Ethics 3
Instrument 3
Procedure 4
Cooling 5
Conditioning 5
Infection prevention 5
Follow-up 6

Endpoints 6
Primary 6
Secondary 6
Tertiary 6
Plan for statistical analyses 7

References 9

Participants – Study organizers 10

Appendices 11
Appendix 1. Grading of oral mucositis 11
Appendix 2. Questionnaire 12
2. I. Evaluation of cooling method (tolerability) 12
2. II. Subjective experiences of OM 18
2. III. General quality of life 20
Appendix 3. Numeric Pain Rating Scale (NRS) 22
Appendix 4. Blood sampling 23

Signature page 24

Flow chart – Myeloma 25
Karolinska Hospital 25
Other Hospitals 26

Flow chart – Lymphoma 27
Karolinska Hospital-BEAM 27
Uppsala University Hospital-BEAC 28

Background

Hematopoietic stem cell transplantation (HSCT) is successfully used for a number of malignant blood diseases. In autologous stem cell transplantation (SCT) the patient's own blood stem cells are used in order to preserve bone marrow function after administration of high doses of cytostatics.

Chemotherapy has many side effects, one of which is oral mucositis (OM). OM is a lesion of the epithelium and the tissue immediately below it in the mouth and throat, affecting up to 80% of patients who receive high doses of cytostatics in preparation for HSCT (1, 2). The lesion of the oral mucosa manifests itself as painful sores in the oral cavity (3) and can require high doses of intravenous morphine for pain relief (4). Furthermore, OM makes food intake difficult, which can lead to undernourishment, weight loss, and impaired quality of life (5).

Today there are few treatment methods intended to prevent the occurrence of OM. An extensive literature search shows that the best-documented preventive the method is cooling of the oral mucous membrane with ice, before, during, and after infusion of cytostatics (6).

Despite well-substantiated documentation, there is limited use of ice in clinical practice. This may be because ice can give rise to shooting pains in the teeth or other discomfort for the patient, leading to lower adherence. In addition, it is very important that the ice is made from water of good quality so that there is no risk of contamination by microorganisms and consequent risk of infections.

To prevent the occurrence of OM, a device called Cooral™ has been designed for oral cooling. Cooral™ consists of a closed duct system with continuously circulating water, shaped and dimensioned to cool the cheeks, lips, mouth floor, tongue, and gums. By offering patients Cooral™ we intend to reduce the incidence of OM but also expect to achieve more even cooling distribution in the oral mucous membrane and better toleration of the cooling temperature compared with ice.

It is of interest to conduct a randomized study comparing Cooral™ and ice as regards tolerability, and the possibility of preventing or relieving OM.

Aim

The primary aim is to study patients with myeloma or lymphoma undergoing autologous SCT, to evaluate whether cooling with Cooral™ compared with ice cubes/crushed ice or ice pop succeeds in reducing the degree of OM according to the Oral Mucositis Assessment Scale, OMAS total.

The secondary aims of the study are to evaluate OMAS total divided according to OMAS ulceration, OMAS erythema, degree of OM according to WHO, tolerability of either cooling method, subjective experience of OM, rating of general quality of life and oral pain, number of days with total parenteral nutrition (TPN), number of hospital days, total dose of opioids, and CRP during time in care.

The tertiary aims of the study are to evaluate weight loss, LPC (leukocyte plasma concentration), number of days until bone marrow response, S-albumin, and body temperature.

Material and Method

Design

An open randomized controlled study with blinded evaluation of OM by a dentist/dental hygienist.

Selection

All patients with myeloma or lymphoma at Uppsala University Hospital and Karolinska Hospital, and patients with myeloma at the University Hospitals in Linköping and Örebro and Rikshospitalet in Oslo who are to undergo autologous SCT will be asked to participate in the study. Information will be given in connection with stem cell apheresis and in material sent to the patient in connection with the invitation letter with information about admission to the ward for autologous SCT. Inclusion in the study will take place after written consent on arrival at the ward to be admitted for autologous SCT. For under-age patients (16–17 years) parents will also be informed and asked if they consent to their children's participation.

Inclusion criteria

I. Patients aged 16 or over diagnosed with myeloma or lymphoma

II. Able to communicate in Swedish/Norwegian

III. Treated with melphalan (myeloma), BEAC/BEAM (lymphoma), before SCT

Exclusion criteria

I. Patients who do not understand oral and written information in Swedish/ Norwegian

II. The patient is taking part in another study which, in the doctor's judgment, can affect the result of this study

III. The patient is receiving post-treatment care at a different hospital than where the stem cell transplant took place and follow-up is not possible

IV. The doctor judges that the patient is for some reason not suitable for the study

Randomization

Randomization will be done by envelope, managed centrally by the study administration in connection with the stem cell apheresis. Each hospital will be given randomization

lists to follow. A total of 180 patients will be randomized to ice or Cooral™ in a proportion of 1:1. Randomization will be stratified with regard to department and diagnosis. Expected number of patients: from Uppsala University Hospital (60), Karolinska Hospital (40), Linköping University Hospital (15) and Örebro University Hospital (15) and Rikshospitalet in Oslo (50).

The patients will undergo balanced randomization with randomly varying block sizes (two, four or six patients) distributed in sequences of one, two, or three experiments and one, two, or three controls. A block can be, for example, "ce" if there are two patients, "ceec" and "eeccec" if there are four or six patients respectively. Each hospital is blinded to the size of the blocks.

Power

With a sample size of 90 patients per group a power of 80% is necessary to discover an average difference of at least 0.42 OMAS units (7). The analysis is based on the standard deviation for OMAS being 1 in both groups, and the use of an independent t-test with a significance level of 5%.

Ethics

All procedures in the study will be carried out in accordance with the Helsinki Declaration of 1964 and its subsequent modifications or comparable ethical norms, as well as ISO 14155:2011, SS-EN ISO 14971:2012. The study has been approved by the local ethical review board in Göteborg (dnr: 586-15).

Instrument

The degree of OM is assessed at eight intraoral locations, in accordance with the Oral Mucositis Assessment Scale (OMAS) (graded 0–3 for ulceration and 0–2 for erythema). 0 corresponds to "normal" while 3 and 2 are "sore >3 cm^2" and "severe erythema" respectively. The assessment generates both an average for OMAS ulceration (0–3) and OMAS erythema (0–2) and a total average OMAS (0–5), which is the mean of both ulceration and erythema.

Besides OMAS, ulceration and erythema are also assessed with the WHO scale (graded 0–4) where 0 is "no mucositis" and 4 is "ulceration, total parenteral nutrition".

Assessment with OMAS and WHO is done by a dentist/dental hygienist, blinded to treatment group, three times a week, for example, Monday, Wednesday, Friday, until discharge or at most day +28. For each day the patient is hospitalized, assessment with WHO is also performed by nurses/assistant nurses who are not blinded to the treatment group.

Furthermore, the patients, after cooling ends, assess the tolerability of the respective cooling method with the aid of a questionnaire developed for the study. The questionnaire is intended to give some idea of any discomfort or side effects the patients feel as a result of the cooling method.

The patients assess their perception of oral problems daily with the aid of specific questions in a diary developed for the study. The questions are intended to give a picture of the effect of OM on the patient's general status.

General quality of life is assessed twice during the study period, before the start of treatment and at discharge, with a validated quality of life instrument.

Oral pain is assessed with a Numeric Pain Rating Scale (NRS) with the extremes graded on a 10-figure scale (0–10) where 0 is "no pain" and 10 is "unbearable pain".

Information about total parenteral nutrition (TPN), number of hospital days, total dose of opioids, weight loss, and body temperature will be retrieved from patient records. Laboratory results of blood tests will be retrieved from each department's register of test results.

The result of the assessments is documented on special CRF (case report forms) for the purpose, referred to in the study as "checklists".

It is only adverse events (AE) and serious adverse events (SAE) that are registered in the questionnaire (appendix 2. I) and diaries (appendix 2. II) and collected during the study.

Procedure

All measurements, with the exception of the patient's subjective assessment of the cooling method, will be registered beginning at admission and will continue until discharge or until day +28. Cytostatic infusion generally starts on the day after admission. Grading of OM according to OMAS and WHO is done three times a week, for example, Monday, Wednesday, Friday, by a dentist/dental hygienist. For each day the patient is hospitalized, assessment with WHO is also performed by nurses/assistant nurses who are not blinded to the treatment group.

Clinical routines differ between different study centers and therefore the number of assessments can be lower during the week of admission, depending on when patients are admitted.

Rating of pain is done 1–2 times daily if the patient is kept on the ward. Alternatively, it is done every other day according to the respective department's routine for outpatient care if the patient is at home or in a home-like environment. In the case of outpatients the subjective rating of pain is noted in the patient documentation through daily telephone contact with the responsible nurse or in accordance with the department's routines.

Assessment of the two cooling methods is performed after completion of ice/Cooral™ cooling in conjunction with cytostatic infusion. Body temperature is registered in the oral cavity in accordance with the department's routines.

Patients who break off the cooling prematurely are followed up in the same way as those who complete the cooling, in accordance with the "intention to treat" principle. If a

patient is unwilling to participate in further follow-up, no new patient will be substituted.

Cooling

Ice
Patients will be provided with ice cubes/crushed ice or ice pop 30 minutes before the start of chemotherapy. As the ice melts, the melted liquid is rinsed around in the mouth to cool as large a part as possible of the oral cavity and throat. To achieve cooling of the hindmost part of the throat, the melted liquid is gurgled for a few seconds before it is swallowed or spat out. When the ice or the pop has melted entirely, yet another selected cooling product is taken immediately. The procedure is repeated until 30 minutes after the termination of the cytostatic infusion. During treatment the patient may if necessary take out the component and rest for a maximum of 5 minutes. Food and drink should be taken either before or after the cytostatic infusion. Cooling continues during conditioning with cytostatics in the treatment schema melphalan (myeloma) and BEAM/BEAC (lymphoma). In lymphoma conditioning cures with a 12-hour infusion time (e.g. Cytarabine) the cooling starts initially 30 minutes before the start of cytostatic treatment and continues 30 minutes after the start of 12-hour cytostatic infusion. Then the patient is provided with ice cubes/crushed ice or ice pop for 30 minutes every 4 hours during the infusion. It is important to end with 30 minutes' cooling of the oral mucous membrane after each completed cytostatics administration.

Cooral™
Before the start of treatment the patient receives clear oral and written instructions on the use of Cooral™ by the nurse responsible for the patient. The patient him/herself is able to administer the intraoral component until it feels comfortable. Then the responsible staff check to ensure that it has good contact with the oral mucous membrane. Cooling begins 30 minutes before the start of chemotherapy and continues during conditioning with cytostatics in the treatment schema melphalan (myeloma) and BEAM/BEAC (lymphoma). Cooling continues until 30 minutes after the termination of the cytostatic infusion. During treatment the patient may if necessary take out the component and replace it again, for a maximum of 5 minutes. Food and drink should thus be taken before or after chemotherapy. In lymphoma conditioning cures with a 12-hour infusion time (e.g. Cytarabine) the cooling starts initially 30 minutes before the start of cytostatic treatment and continues 30 minutes after the start of 12-hour cytostatic infusion. Then the patient is provided with Cooral™ for 30 minutes every 4 hours during the infusion. It is important to end with 30 minutes' cooling of the oral mucous membrane after each completed cytostatics administration.

Conditioning
All administration of cytostatics is intravenous (i.v.)

Infection prevention

I. Odontological decontamination
II. Prevention of infection and fungus in accordance with the department's routines

Follow-up

I. All patients will be followed up day −1/0 (start of cytostatic infusion) and then daily until discharge or at most 28 days

Endpoints

Primary

I. Degree of OM according to OMAS total?

Secondary

I. OMAS ulceration and OMAS erythema?

II. Degree of OM according to WHO?

III. Tolerability

    A. Discomfort from cold
    B. Other side effects

IV. Subjective experience of OM measured with a diary constructed for the study

V. Rating of general quality of life measured by a validated quality of life instrument (FACT-G).

VI. Rating of oral pain measured with a Numeric Pain Rating Scale (NRS)

VII. Number of days with TPN

VIII. Number of hospital days

IX. Total dose of opioid converted to morphine (mg)

X. CRP level during time in care

Tertiary

I. Weight loss in kilograms (kg) during time in care

II. LPC according to the department's routines during time in care

III. Number of days until bone marrow results (neutrophils > 0.5)

IV. S-Albumin according to the hospital's routines during time in care

V. Body temperature

Plan for statistical analyses

All analyses are at population level: Intention-to-treat.

Analysis of the primary variable
The primary endpoint is peak OMAS (total). i.e. the highest measured OMAS total during the time in care. The primary variable is studied in a multiple linear regression model. Fixed explanatory variables are: treatment group, type of cancer, and center. An initial model also includes interaction between treatment and type of cancer and interaction between treatment and center. If the interaction effects are not significant, these are excluded from the final model. The significance level used is 5%.

Analysis of the secondary variable
OMAS ulceration and OMAS erythema indices, analyzed in the same way as peak OMAS (total), i.e., peak value is used as a target variable in a multiple regression model. The same explanatory variables are used as in the final model for peak OMAS (total).

Incidence of OM (grades 1–4 according to WHO) and incidence of severe OM (grades 3–4 according to WHO) are analyzed with the aid of logistic regression with the same explanatory variables as in the final model for peak OMAS (total). Significance level 5%.

Tolerability. Incidence of problems (grades 1–3) and severe problems (grades 4–7) are analyzed in the same way as the incidence of OM and severe OM. Significance level 5%.

Subjective ratings of OM, general quality of life and oral pain are analyzed non-parametrically, above all with the help of Mann-Whitney's U test.

Quantitative data such as number of days with TPN, number of hospital days, total dose of opioids, and CRP are analyzed with independent t-test, or with Mann-Whitney's U test if the observed data material shows a significant skew. Descriptive statistics and explorative analysis will be used to study any differences between centers.

Analysis of the tertiary variables
Weight loss, LPC, number of days until bone marrow response, S-Albumin and body temperature are analyzed with independent t-test, or with Mann-Whitney U's test if the observed data material shows a significant skew. Descriptive statistics and explorative analysis will be used to study any differences between centers.

Missing data
In the absence of OMAS total or WHO performed by a dentist/dental hygienist after the treatment, WHO performed by nurses/assistant nurses is used instead. WHO as a substitute for OMAS total is translated to OMAS total (8). In the final analysis it is primarily a dentist's assessment that is used. For OMAS subindex the highest value is used as peak OMAS subindex if there is at least one OMAS subindex after treatment. If there is no OMAS subindex after treatment, the patient's baseline is used as peak OMAS subindex.

For other secondary/tertiary variables the strategy is to use the mean value of the preceding and following value. If the preceding value is missing, the following value is used. If the following value is missing, the technique used is last value carried forward.

Data Monitoring Committee
To protect the patients' safety during the trial, the results will be monitored by a Data Monitoring Committee (DMC), consisting of an experienced biostatistician and a clinician with long experience of clinical trials. Both are independent of the sponsor and will provide an impartial recommendation for the continuation of the study.

An interim analysis will be performed when the results for the first hundred patients are available. The results will be communicated only to the members of the Data Monitoring Committee, who will then, on the basis of the results, recommend the sponsor either to continue or to stop the trial. The study may be terminated early if serious side effects arise or if the difference in effect between the two treatments exceeds what is deemed clinically relevant. Separate working instructions will be provided as a "charter" to the Data Monitoring Committee. A conservative stopping rule according to the O'Brien-Fleming boundary will be applied to minimize the effect of the interim analysis on the statistical strength at the end of the trial.

The ethical review board will be informed if the study is terminated prematurely. Inclusion of patients will continue during the time the interim analysis is being performed.

References

1. PETERSON DE, BENSADOUN RJ, ROILA F et al. Management of oral and gastrointestinal mucositis: ESMO Clinical Practice Guidelines. Ann Oncol 2011; 22 Suppl 6: vi78-84.
2. NAIDU MU, RAMANA GV, RANI PU et al. Chemotherapy-induced and/or radiation therapy-induced oral mucositis--complicating the treatment of cancer. Neoplasia 2004; 6: 423-31.
3. MCGUIRE DB, ALTOMONTE V, PETERSON DE et al. Patterns of mucositis and pain in patients receiving preparative chemotherapy and bone marrow transplantation. Oncology nursing forum 1993; 20: 1493-502.
4. RUBENSTEIN EB, PETERSON DE, SCHUBERT M et al. Clinical practice guidelines for the prevention and treatment of cancer therapy-induced oral and gastrointestinal mucositis. Cancer 2004; 100: 2026-46.
5. ELTING LS, KEEFE DM, SONIS ST et al. Patient-reported measurements of oral mucositis in head and neck cancer patients treated with radiotherapy with or without chemotherapy: demonstration of increased frequency, severity, resistance to palliation, and impact on quality of life. Cancer 2008; 113: 2704-13.
6. LALLA RV, BOWEN J, BARASCH A et al. MASCC/ISOO clinical practice guidelines for the management of mucositis secondary to cancer therapy. Cancer 2014; 120: 1453-61.
7. PETERSON DE, BARKER NP, AKHMADULLINA LI et al. Phase II, randomized, double-blind, placebo-controlled study of recombinant human intestinal trefoil factor oral spray for prevention of oral mucositis in patients with colorectal cancer who are receiving fluorouracil-based chemotherapy. J Clin Oncol 2009; 27: 4333-8.
8. LEGERT KG, REMBERGER M, RINGDEN O et al. Reduced intensity conditioning and oral care measures prevent oral mucositis and reduces days of hospitalization in allogeneic stem cell transplantation recipients. Support Care Cancer 2014; 22: 2133-40.

Participants – Study organizers

Oral Medicine Clinic, Department of Odontology
Mats Jontell, Professor, Senior dental officer (project leader)
Division of Oral Medicine and Pathology
E-mail: mats.jontell@odontologi.gu.se

Java Walladbegi, Doctoral student, Dentist
Division of Oral Medicine and Pathology
E-mail: java.walladbegi@odontologi.gu.se

Uppsala University Hospital:
Anncarin Svanberg, MD, Nurse
Section for Hematology & Coagulation
E-mail: ann-carin.svanberg@akademiska.se

PI: Gunnar Larfors, MD, Senior physician
Section for Hematology & Coagulation
E-mail: gunnar.larfors@akademiska.se

Karolinska University Hospital
PI: Martin Jädersten, MD PhD, Överläkare
Hematology Center, M54
E-post: per.broliden@karolinska.se

Coordinating PI: Karin Garming Legert PhD, Dentist
Section for Orofacial diagnosis and surgery
E-mail: karin.garming.legert@ki.se

Linköping University Hospital:
PI: Franz Rommel, Senior physician
Section for Hematology
E-mail: franz.rommel@regionostergotland.se

Örebro University Hospital:
PI: Erik Ahlstrand, Senior physician
Section for Hematology
E-mail: erik.ahlstrand@regionorebrolan.se

Rikshospitalet in Oslo:
PI: Fredrik Schjesvold, Senior physician
Section for Hematology
E-post: fredrikschjesvold@gmail.com

Appendices

Appendix 1. Grading of oral mucositis

OMAS ulceration

0	1	2	3
Normal	< 1 cm^3	1–3 cm^3	> 3 cm^3

OMAS erythema

0	1	2
Normal	Slight erythema	Severe erythema

WHO grading of oral mucositis

Grade 0	Grade 1	Grade 2	Grade 3	Grade 4
No mucositis	Pain/erythema	Erythema/Sores<br>Can eat and drink	Sores<br>Can only drink	Sores<br>Total parenteral nutrition

Appendix 2. Questionnaire

2. I. Evaluation of cooling method (tolerability)

Questionnaire about Cooral™ cooling

1. Did you manage to have Cooral™ in your mouth the whole cooling time?

☐ Yes (skip to question 4)

☐ No

2. Roughly how long did you have Cooral™ in your mouth?

☐ 1–20 minutes

☐ 21–40 minutes

☐ 41–60 minutes

☐ 61–80 minutes

☐ 81–100 minutes

☐ >100 minutes but not the full time

3. Which of the following was the reason? Mark the letter or letters.

A ☐ I got cold

B ☐ I became numb

C ☐ It tasted bad

D ☐ I got a headache

E ☐ Shooting pains in my teeth

F ☐ My mouth got sore

G ☐ Poor fit

H ☐ I felt nauseous

I ☐ I felt I needed to vomit

J ☐ It was difficult to swallow

K ☐ It chafed

L ☐ Other......................................

4. Was it unpleasant to have Cooral™ in your mouth?

☐ No, not at all (skip to question 6)

☐ No, hardly at all

☐ Yes, a little

☐ Yes, very much so

5. If you experienced some form of discomfort, in what way was it unpleasant? (several alternatives may be chosen)

A ☐ I got cold

B ☐ I became numb

C ☐ It tasted bad

D ☐ I got a headache

E ☐ Shooting pains in my teeth

F ☐ My mouth got sore

G ☐ Poor fit

H ☐ I felt nauseous

I ☐ I felt I needed to vomit

J ☐ It was difficult to swallow

K ☐ It chafed

L ☐ Other.......................................

6. Did Cooral™ limit your ability to do something else during the time?

☐ No, not at all

☐ No, not very much

☐ Yes, a little

☐ Yes, very much so

7. Other viewpoints...

8. How painful was the cooling of the oral mucous membrane?

1. ☐ Not at all painful
2. ☐ Slightly painful
3. ☐ Rather painful
4. ☐ Painful
5. ☐ Very painful
6. ☐ Very, very painful
7. ☐ Extremely painful, was forced to break off cooling before the end

Questionnaire about cooling with ice / crushed ice / ice pop

1. Which cooling alternative did you use?

☐ Ice

☐ Crushed ice

☐ Ice pop

2. Did you manage to have the ice in your mouth the whole cooling time?

☐ Yes (skip to question 5)

☐ No

3. Roughly how long did you have ice in your mouth?

☐ 1–20 minutes

☐ 21–40 minutes

☐ 41–60 minutes

☐ 61–80 minutes

☐ 81–100 minutes

☐ >100 minutes but not the full time

4. Which of the following was the reason? Mark the letter or letters.

A ☐ I got cold

B ☐ I became numb

C ☐ It tasted bad

D ☐ I got a headache

E ☐ Shooting pains in my teeth

F ☐ My mouth got sore

G ☐ I felt nauseous

H ☐ I felt I needed to vomit

I ☐ It was difficult to swallow

J ☐ Other.......................................

5. Was it unpleasant to have the ice in your mouth?

☐ No, not at all (skip to question 7)

☐ No, hardly at all

☐ Yes, a little

☐ Yes, very much so

6. If you experienced some form of discomfort, in what way was it unpleasant? (several alternatives may be chosen)

A ☐ I got cold

B ☐ I became numb

C ☐ It tasted bad

D ☐ I got a headache

E ☐ Shooting pains in my teeth

F ☐ My mouth got sore

G ☐ I felt nauseous

H ☐ I felt I needed to vomit

I ☐ It was difficult to swallow

J ☐ Other.......................................

7. Did the ice limit your ability to do something else during the time?

☐ No, not at all

☐ No, not very much

☐ Yes, a little

☐ Yes, very much so

8. Other viewpoints...

9. How painful was the cooling of the oral mucous membrane?

1. ☐ Not at all painful
2. ☐ Slightly painful
3. ☐ Rather painful
4. ☐ Painful
5. ☐ Very painful
6. ☐ Very, very painful
7. ☐ Extremely painful, was forced to break off cooling before the end

2. II. Subjective experiences of OM

Diary

1. Pain in the mouth:

Please circle a figure on the scale below to show how severe the pain in your mouth is NOW

0 1 2 3 4 5 6 7 8 9 10

No pain **Unbearable pain**

2. Impact on taste:

Please circle a figure on the scale below to show the change in your sense of taste NOW

0 1 2 3 4 5 6 7 8 9 10

No change in taste **Very noticeable change in taste**

3. Impact on smell:

Please circle a figure on the scale below to show the change in your sense of smell NOW

0 1 2 3 4 5 6 7 8 9 10

No change in smell **Very noticeable change in smell**

4. Please circle a figure on the scale below to show the effect on the your ability to perform the acts below (**0**=no effect; **10**=maximum effect)

A. Swallow

0 1 2 3 4 5 6 7 8 9 10

B. Drink

0 1 2 3 4 5 6 7 8 9 10

C. Eat

0 1 2 3 4 5 6 7 8 9 10

D. Speak

0 1 2 3 4 5 6 7 8 9 10

E. Sleep

0 1 2 3 4 5 6 7 8 9 10

5. Other viewpoints you would like us to know.

2. III. General quality of life

FACT-G (Version 4)

Below is a list of statements that other people with your illness have said are important. **Please circle or mark one number per line to indicate your response as it applies to the past 7 days.**

	PHYSICAL WELL-BEING	Not at all	A little bit	Some-what	Quite a bit	Very much
GP1	I have a lack of energy	0	1	2	3	4
GP2	I have nausea	0	1	2	3	4
GP3	Because of my physical condition, I have trouble meeting the needs of my family	0	1	2	3	4
GP4	I have pain	0	1	2	3	4
GP5	I am bothered by side effects of treatment	0	1	2	3	4
GP6	I feel ill	0	1	2	3	4
GP7	I am forced to spend time in bed	0	1	2	3	4

	SOCIAL/FAMILY WELL-BEING	Not at all	A little bit	Some-what	Quite a bit	Very much
GS1	I feel close to my friends	0	1	2	3	4
GS2	I get emotional support from my family	0	1	2	3	4
GS3	I get support from my friends	0	1	2	3	4
GS4	My family has accepted my illness	0	1	2	3	4
GS5	I am satisfied with family communication about my illness	0	1	2	3	4
GS6	I feel close to my partner (or the person who is my main support)	0	1	2	3	4
Q1	*Regardless of your current level of sexual activity, please answer the following question. If you prefer not to answer it, please mark this box ☐ and go to the next section.*					
GS7	I am satisfied with my sex life	0	1	2	3	4

FACT-G (Version 4)

Please circle or mark one number per line to indicate your response as it applies to the past 7 days.

	EMOTIONAL WELL-BEING	Not at all	A little bit	Some-what	Quite a bit	Very much
GE1	I feel sad	0	1	2	3	4
GE2	I am satisfied with how I am coping with my illness	0	1	2	3	4
GE3	I am losing hope in the fight against my illness	0	1	2	3	4
GE4	I feel nervous	0	1	2	3	4
GE5	I worry about dying	0	1	2	3	4
GE6	I worry that my condition will get worse	0	1	2	3	4

	FUNCTIONAL WELL-BEING	Not at all	A little bit	Some-what	Quite a bit	Very much
GF1	I am able to work (include work at home)	0	1	2	3	4
GF2	My work (include work at home) is fulfilling	0	1	2	3	4
GF3	I am able to enjoy life	0	1	2	3	4
GF4	I have accepted my illness	0	1	2	3	4
GF5	I am sleeping well	0	1	2	3	4
GF6	I am enjoying the things I usually do for fun	0	1	2	3	4
GF7	I am content with the quality of my life right now	0	1	2	3	4

Appendix 3. Numeric Pain Rating Scale (NRS)

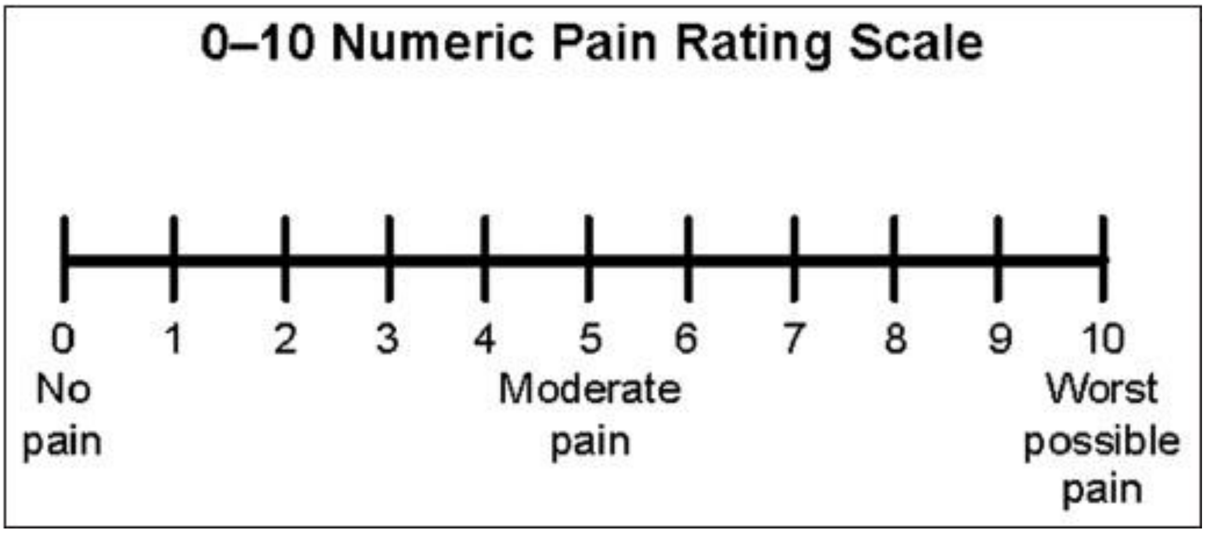

Appendix 4. Blood sampling

AKADEMISKA SJUKHUSET

Sektionen för Hematologi

Allogen/autolog/poliklin HSCT, AL ind/rem - veckoprovtagning rutin

ID: XD355-2 sida 2 (4) Giltigt i 2 år från: 2014-01-03

2. Autolog HSCT samt akut leukemi (remission) – veckoprov-tagning

Översikt

**Klinisk kemi och farmakologi (KKF)**	Mån	Tis	Ons	Tors	Fre	Lör	Sön
Blodstatus *B-Neutrofila tas endast vid LPK (totalvita) ≥ 0,7*<br>P-CRP	X		X		X		X
Elektrolytstatus *(Na, K, krea) Tas dagl. vid cytokur och TPN*<br>P-Glukos *Fasteprov*	X		X		X		
Leverstatus 50C *(P-ALAT, P-ASAT, P-Bilirubin, P-alk.fosfatas, P-LD)*<br>P-Albumin, P-PK	X						

Om möjligt:

- stäng av pågående infusion/er i minst 5 min innan blodprov tas.
- bör infusion med glukos stängas av 1 timme innan prov för P-Glukos.
- bör det ha gått minst 8 timmar sedan TPN-infusion då blodprover tas.

(Källa: Akademiska laboratoriet)

- För **rutinmässiga odlingar vid symtom** samt provtagning för blododling, se Odlingar, rutinmässiga, sektionen för Hematologi XD237 i Kvalitetshandboken.

Provpaket i Cosmic (genvägar)

Måndag: KKF: *Vecko (AL/ind/rem/SCT(Med)*
Om aktuellt, lägg till B-Neutrofila.

Onsdag: KKF: *Ons/fre (allo/AL/ind/rem/SCT)Med)*

Fredag: KKF: *Ons/fre (allo/AL/ind/rem/SCT)Med)*

Signature page

Signature
(sponsor)

Printed name

Date

Signature
(trial coordinator)

Printed name

Date

Flow chart – Myeloma
Karolinska Hospital

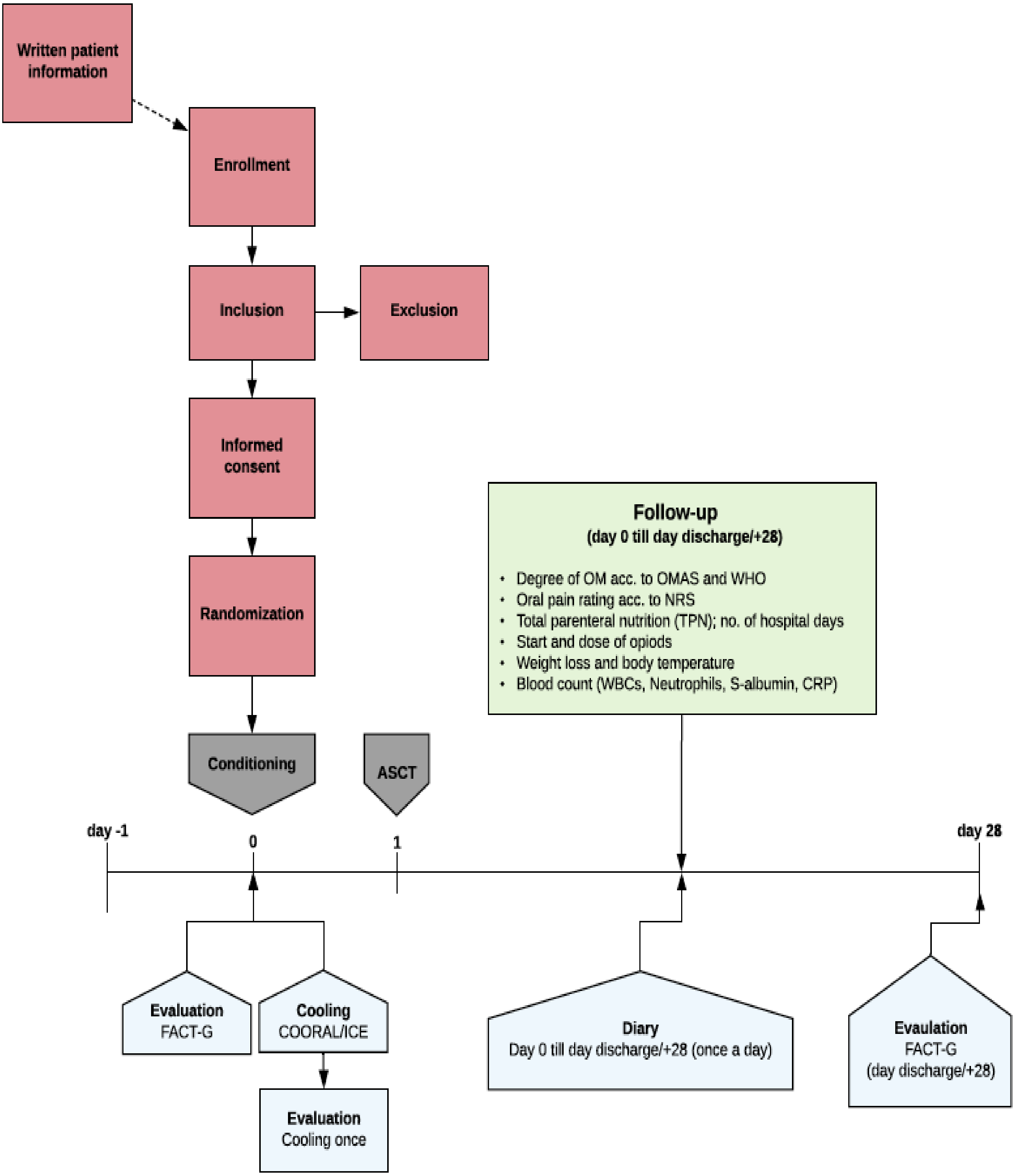

Flow chart – Myeloma

Other Hospitals

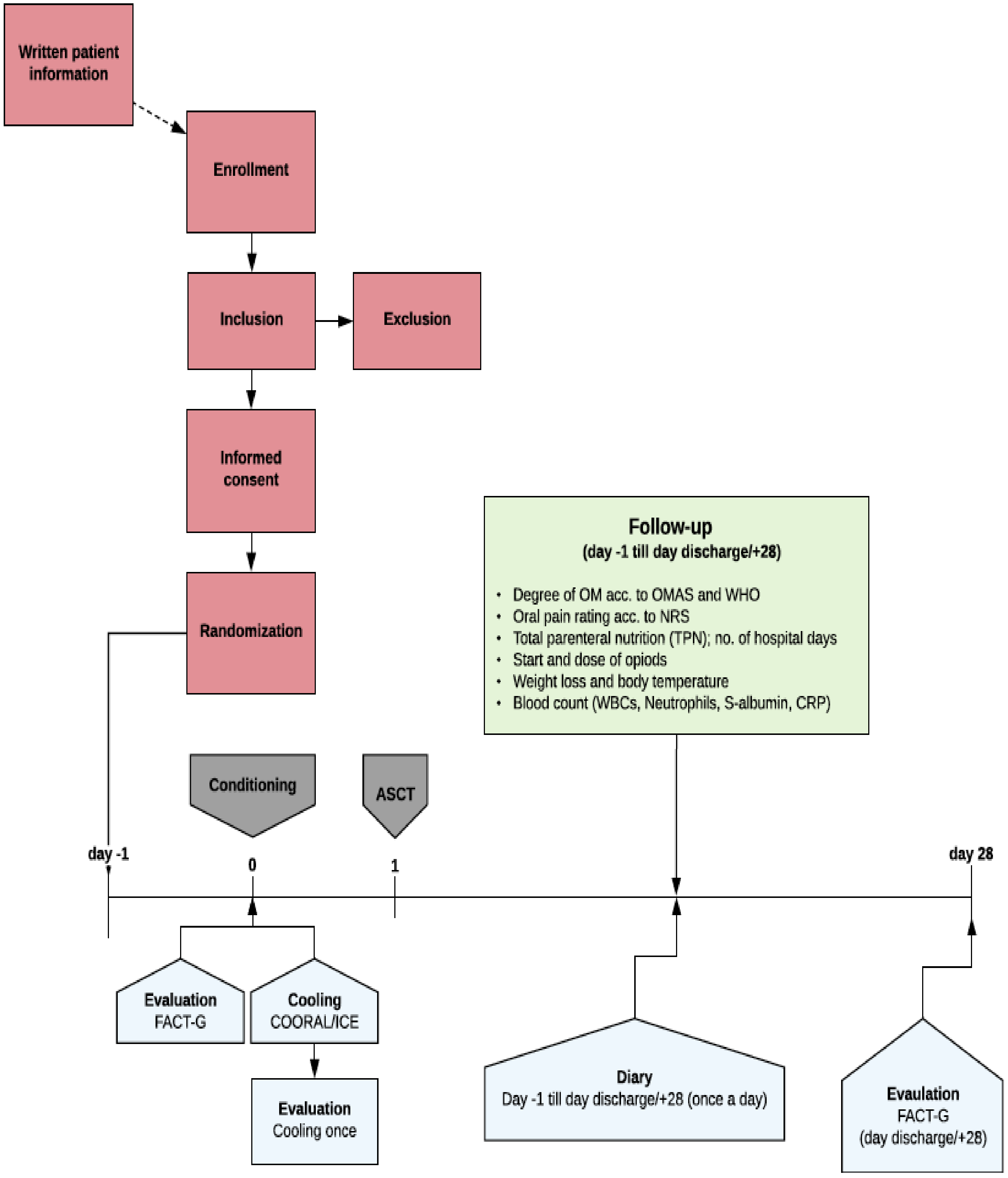

Flow chart – Lymphoma
Karolinska Hospital-BEAM

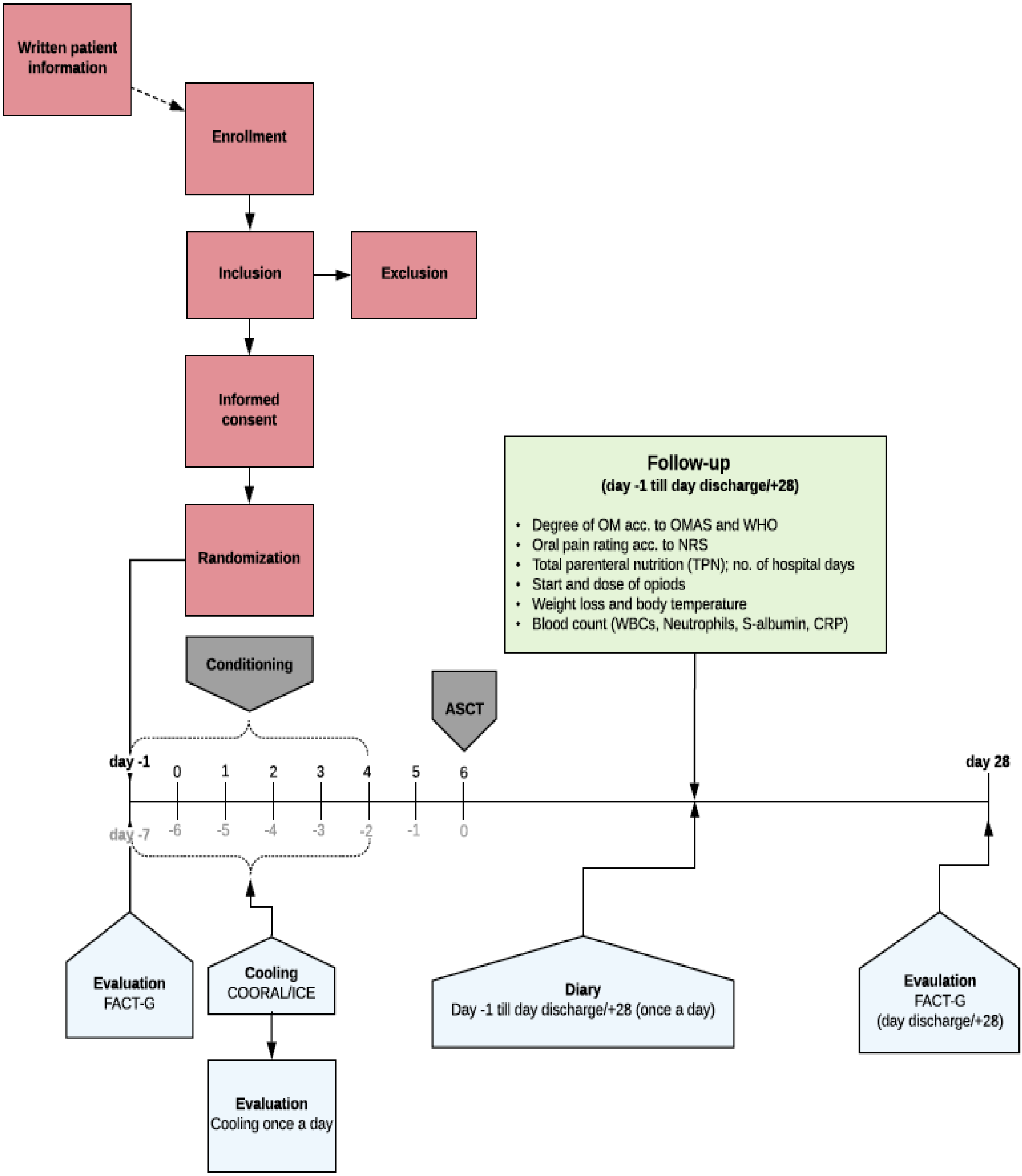

Flow chart – Lymphoma
Uppsala University Hospital-BEAC

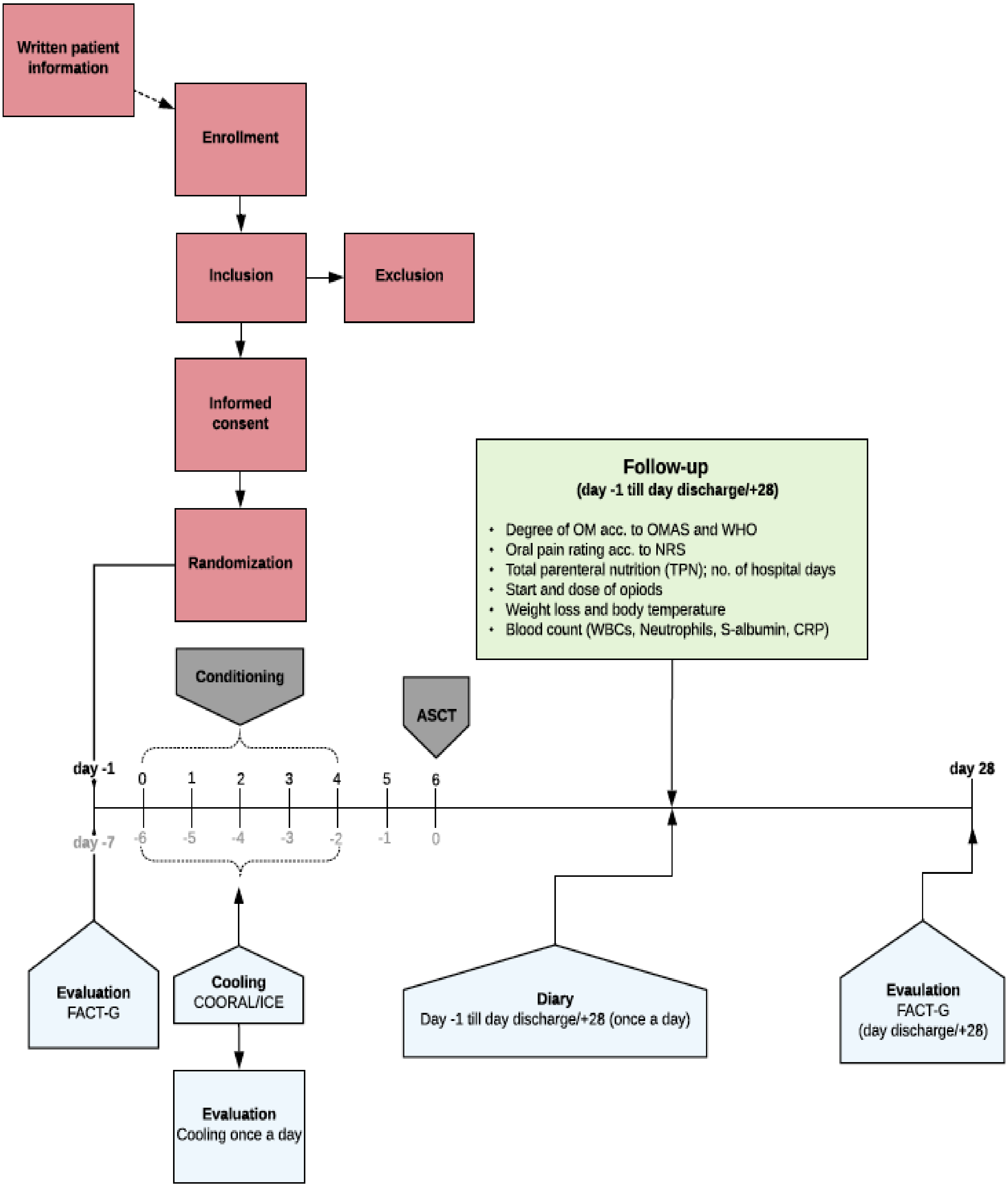

Supplement: Supplementary file 1 — Supplement 1 [file 41409_2021_1512_MOESM1_ESM.pdf]
