# Supplementary material for: Efficacy of a novel device for cryoprevention of oral mucositis: a randomized, blinded, multicenter, parallel group, phase 3 trial
Source: Bone Marrow Transplant. 2021 Nov 3;57(2):191–7. doi: 10.1038/s41409-021-01512-6 (PMC8821013; doi:10.1038/s41409-021-01512-6)
Supplement: Supplementary file 2 — Supplement 2 [file 41409_2021_1512_MOESM2_ESM.pdf]

Supplement 2

Conditioning high-dose chemotherapy, infection prophylaxis in conjunction with cytopenia, and supportive care including standard oral care were administered according to institutional practice. Patients with multiple myeloma were conditioned with a single-dose intravenous Melphalan 140 mg/m^2 or 200 mg/m^2 on day -1, i.e., 24 hours before ASCT. Lymphoma patients were conditioned with either of the two high-dose chemotherapy regimens BEAC or BEAM. BEAC was administered according to the following figure: Day -6: carmustine 300 mg/m^2 and etoposide 100 mg/m^2; Day -5 to -3: etoposide 200 mg/ m^2, cytarabine 200 mg/m^2 and cyclophosphamide 35 mg/kg; Day -2: etoposide 100 mg/m^2, cytarabine 200 mg/m^2 and cyclophosphamide 35 mg/kg; Day -1: Recovery; Day 0: ASCT. The corresponding figure for BEAM was: Day -7: carmustine 300 mg/m^2; Day -6 to -3: etoposide 200 mg/m^2 and cytarabine 400 mg/m^2; Day -2: melphalan 140 mg/m^2; Day -1: Recovery; Day 0: ASCT. All patients received autologous multipotent hematopoietic stem cells derived from peripheral-blood, collected after routine mobilization by means of chemotherapy and granulocyte–colony-stimulating factor (G-CSF; filgrastim), with a dose of ≥ 2 x 10^6 CD34+ cells/kg. Following ASCT, filgrastim or lipegfilgrastim was optionally administered according to the clinical routines established at each study site.
