# Supplementary material for: Efficacy of a novel device for cryoprevention of oral mucositis: a randomized, blinded, multicenter, parallel group, phase 3 trial
Source: Bone Marrow Transplant. 2021 Nov 3;57(2):191–7. doi: 10.1038/s41409-021-01512-6 (PMC8821013; doi:10.1038/s41409-021-01512-6)
Supplement: Supplementary file 3 — Supplement 3 [file 41409_2021_1512_MOESM3_ESM.pdf]

Supplement 3

OMAS – Oral Mucositis Assessment Scale

Circle:

Location	Ulceration / pseudomembrane				Erythema		
Upper lip	0	1	2	3	0	1	2
Lower lip	0	1	2	3	0	1	2
Right cheek	0	1	2	3	0	1	2
Left cheek	0	1	2	3	0	1	2
Right ventral and lateral tongue	0	1	2	3	0	1	2
Left ventral and lateral tongue	0	1	2	3	0	1	2
Floor of the mouth	0	1	2	3	0	1	2
Palatal mucosae/gingiva	0	1	2	3	0	1	2

Ulceration / pseudomembrane:
0 = no lesion
1 = < 1 cm^2
2 = 1 – 3 cm^2
3 = > 3 cm^2

Erythema:
0 = none
1 = mild
2 = severe
