# Supplementary material for: Efficacy of a novel device for cryoprevention of oral mucositis: a randomized, blinded, multicenter, parallel group, phase 3 trial
Source: Bone Marrow Transplant. 2021 Nov 3;57(2):191–7. doi: 10.1038/s41409-021-01512-6 (PMC8821013; doi:10.1038/s41409-021-01512-6)
Supplement: Supplementary file 4 — Supplement 4 [file 41409_2021_1512_MOESM4_ESM.pdf]

Questionnaire about cooling with ice chips (IC)

1. Did you manage to have the IC in your mouth the whole cooling time?

- ☐ Yes (skip to question 4)
- ☐ No

2. Roughly how long did you have the IC in your mouth?

- ☐ 1–20 minutes
- ☐ 21–40 minutes
- ☐ 41–60 minutes
- ☐ 61–80 minutes
- ☐ 81–100 minutes
- ☐ >100 minutes but not the full time

3. Which of the following was the reason? Mark one or more alternatives.

- ☐ Chills
- ☐ Numbness
- ☐ Bad taste
- ☐ Headache
- ☐ Teeth sensation
- ☐ Oral soreness
- ☐ Nausea
- ☐ Vomiting sensation
- ☐ Difficulties swallowing
- ☐ Other discomforts..

4. Was it unpleasant to have the IC in your mouth?

- ☐ No, not at all (skip to question 6)
- ☐ No, hardly at all
- ☐ Yes, a little
- ☐ Yes, very much so

5. If you experienced some form of discomfort, in what way was it unpleasant? (several alternatives may be chosen)

- ☐ Chills
- ☐ Numbness
- ☐ Bad taste
- ☐ Headache
- ☐ Teeth sensations
- ☐ Oral soreness
- ☐ Nausea
- ☐ Vomiting sensation
- ☐ Difficulties swallowing
- ☐ Other discomforts..

6. Did the IC limit your ability to do something else during the time?

- ☐ No, not at all
- ☐ No, not very much
- ☐ Yes, a little
- ☐ Yes, very much so

7. Other viewpoints..

8. How painful was the cooling of the oral mucous membrane?

- ☐ Not at all painful
- ☐ Slightly painful
- ☐ Rather painful
- ☐ Painful
- ☐ Very painful
- ☐ Very, very painful
- ☐ Extremely painful, was forced to break off cooling before the end

Questionnaire about cooling with intraoral cooling device (ICD)

1. Did you manage to have ICD in your mouth the whole cooling session?

- ☐ Yes (skip to question 4)
- ☐ No

2. Roughly how long did you have the ICD in your mouth?

- ☐ 1–20 minutes
- ☐ 21–40 minutes
- ☐ 41–60 minutes
- ☐ 61–80 minutes
- ☐ 81–100 minutes
- ☐ >100 minutes but not the full time

3. Which of the following was the reason? Mark one or more alternatives.

- ☐ Chills
- ☐ Numbness
- ☐ Bad taste
- ☐ Headache
- ☐ Teeth sensations
- ☐ Oral soreness
- ☐ Poor fit
- ☐ Nausea
- ☐ Vomiting sensation
- ☐ Difficulties swallowing
- ☐ Rubbing discomfort
- ☐ Other discomforts..

4. Was it unpleasant to have the ICD in your mouth?

- ☐ No, not at all (skip to question 6)
- ☐ No, hardly at all
- ☐ Yes, a little
- ☐ Yes, very much so

5. If you experienced any form of discomfort, in what way was it unpleasant? (several alternatives may be chosen)

- ☐ Chills
- ☐ Numbness
- ☐ Bad taste
- ☐ Headache
- ☐ Teeth sensations
- ☐ Oral soreness
- ☐ Poor fit
- ☐ Nausea
- ☐ Vomiting sensation
- ☐ Difficulties swallowing
- ☐ Rubbing discomfort
- ☐ Other discomforts...

6. Did the ICD limit your ability to do something else during the time?

- ☐ No, not at all
- ☐ No, not very much
- ☐ Yes, a little
- ☐ Yes, very much so

7. Other viewpoints...

8. How painful was the cooling of the oral mucous membrane?

- ☐ Not at all painful
- ☐ Slightly painful
- ☐ Rather painful
- ☐ Painful
- ☐ Very painful
- ☐ Very, very painful
- ☐ Extremely painful, was forced to break off cooling before the end
