# Supplementary material for: Efficacy of a novel device for cryoprevention of oral mucositis: a randomized, blinded, multicenter, parallel group, phase 3 trial
Source: Bone Marrow Transplant. 2021 Nov 3;57(2):191–7. doi: 10.1038/s41409-021-01512-6 (PMC8821013; doi:10.1038/s41409-021-01512-6)
Supplement: Supplementary file 5 — Supplement 5 [file 41409_2021_1512_MOESM5_ESM.pdf]

FACT-G (Version 4)

Below is a list of statements that other people with your illness have said are important. **Please circle or mark one number per line to indicate your response as it applies to the past 7 days.**

	**PHYSICAL WELL-BEING**	**Not at all**	**A little bit**	**Some-what**	**Quite a bit**	**Very much**
GP1	I have a lack of energy	0	1	2	3	4
GP2	I have nausea	0	1	2	3	4
GP3	Because of my physical condition, I have trouble meeting the needs of my family	0	1	2	3	4
GP4	I have pain	0	1	2	3	4
GP5	I am bothered by side effects of treatment	0	1	2	3	4
GP6	I feel ill	0	1	2	3	4
GP7	I am forced to spend time in bed	0	1	2	3	4

	**SOCIAL/FAMILY WELL-BEING**	**Not at all**	**A little bit**	**Some-what**	**Quite a bit**	**Very much**
GS1	I feel close to my friends	0	1	2	3	4
GS2	I get emotional support from my family	0	1	2	3	4
GS3	I get support from my friends	0	1	2	3	4
GS4	My family has accepted my illness	0	1	2	3	4
GS5	I am satisfied with family communication about my illness	0	1	2	3	4
GS6	I feel close to my partner (or the person who is my main support)	0	1	2	3	4
Q1	*Regardless of your current level of sexual activity, please answer the following question. If you prefer not to answer it, please mark this box* ☐ *and go to the next section.*					
GS7	I am satisfied with my sex life	0	1	2	3	4

FACT-G (Version 4)

Please circle or mark one number per line to indicate your response as it applies to the past 7 days.

	EMOTIONAL WELL-BEING	Not at all	A little bit	Some-what	Quite a bit	Very much
GE1	I feel sad	0	1	2	3	4
GE2	I am satisfied with how I am coping with my illness	0	1	2	3	4
GE3	I am losing hope in the fight against my illness	0	1	2	3	4
GE4	I feel nervous	0	1	2	3	4
GE5	I worry about dying	0	1	2	3	4
GE6	I worry that my condition will get worse	0	1	2	3	4

	FUNCTIONAL WELL-BEING	Not at all	A little bit	Some-what	Quite a bit	Very much
GF1	I am able to work (include work at home)	0	1	2	3	4
GF2	My work (include work at home) is fulfilling	0	1	2	3	4
GF3	I am able to enjoy life	0	1	2	3	4
GF4	I have accepted my illness	0	1	2	3	4
GF5	I am sleeping well	0	1	2	3	4
GF6	I am enjoying the things I usually do for fun	0	1	2	3	4
GF7	I am content with the quality of my life right now	0	1	2	3	4
